# Macrophages in sepsis-induced acute lung injury: exosomal modulation and therapeutic potential

**DOI:** 10.3389/fimmu.2024.1518008

**Published:** 2025-01-07

**Authors:** Kaiying Lv, Qun Liang

**Affiliations:** ^1^ Graduate School, Heilongjiang University of Chinese Medicine, Harbin, China; ^2^ Department of Critical Care Medicine, First Affiliated Hospital of Heilongjiang University of Chinese Medicine, Harbin, China

**Keywords:** macrophages, sepsis-induced acute lung injury, exosomes, inflammation, therapy abbreviations

## Abstract

Sepsis-induced acute lung injury (ALI) remains a leading cause of mortality in critically ill patients. Macrophages, key modulators of immune responses, play a dual role in both promoting and resolving inflammation. Exosomes, small extracellular vesicles released by various cells, carry bioactive molecules that influence macrophage polarization and immune responses. Emerging researchers have identified exosomes as crucial mediators that modulate macrophage activity during sepsis-induced ALI. This review explores the role of exosomes in modulating macrophage functions, focusing on the cellular interactions within the lung microenvironment and their potential as therapeutic targets. It highlights the regulation of macrophages by exosomes derived from pathogenic germs, neutrophils, alveolar epithelial cells, and mesenchymal stromal cells. By understanding these mechanisms, it aims to uncover innovative therapeutic strategies for sepsis-induced ALI.

## 1 Introduction

Sepsis-induced acute lung injury (ALI) is a life-threatening condition characterized by severe pulmonary inflammation, increased vascular permeability, alveolar damage, and respiratory dysfunction (1). The overwhelming inflammation often leads to the development of acute respiratory distress syndrome, a condition with high morbidity and mortality rates, especially in critically ill patients (2). Despite significant advances in the management of sepsis-induced ALI, effective therapeutic interventions targeting the underlying pathophysiology of ALI remain elusive (3), and mortality rates remain high, ranging between 30-50% (3, 4). As key effectors of the innate immune system, macrophages are involved in pathogen recognition, cytokine production, and tissue repair. They display remarkable plasticity, being capable of adopting different functional states depending on the signals from their environment (5, 6). In sepsis-induced ALI, macrophages can polarize into M1 macrophages, which are pro-inflammatory and responsible for initiating and sustaining the inflammatory response, or M2 macrophages, which are anti-inflammatory and involved in resolving inflammation and promoting tissue repair (7, 8). The balance between these two phenotypes is critical in determining the outcome of ALI, with excessive M1 activation leading to tissue damage and fibrosis, while insufficient M2 activation impairs the resolution of inflammation and tissue regeneration (9, 10).

Exosomes are a kind of small extracellular vesicles (30-150 nm) that are released by a wide variety of cell types. These vesicles carry proteins, lipids, and nucleic acids like mRNAs, microRNAs (miRNAs), and long non-coding RNAs (lncRNAs), which can be transferred into target cells to influence their function (11, 12). Exosomes have been shown to play a pivotal role in intercellular communication and the regulation of immune responses, making them function as key players in the pathophysiology of sepsis-induced ALI. By transferring bioactive molecules between cells, exosomes can modulate macrophage polarization, promoting either pro-inflammatory M1 responses or anti-inflammatory M2 responses depending on their cargo and cell sources (13, 14). Exosomes have been implicated in several crucial processes, including the amplification of inflammation, modulation of immune responses, and facilitation of tissue repair. For example, exosomes derived from neutrophils, epithelial cells, or pathogens can promote M1 macrophage polarization, thereby exacerbating lung inflammation and injury (15–17). Whereas, exosomes released by mesenchymal stromal cells (MSCs) can promote M2 macrophage polarization, aiding in the resolution of inflammation and tissue regeneration (18). The ability of exosomes to selectively influence macrophage function has made them an attractive target for therapeutic interventions in sepsis-induced ALI, offering the potential to modulate immune responses and alleviate lung injury.

This review concisely summarizes the role of macrophages in sepsis-induced ALI and the biogenesis and molecular composition of exosomes, focusing on how exosomes derived from various cell sources modulate macrophage activity and influence disease progression. It also explores the potential of exosomes as therapeutic agents, highlighting recent advances in exosome-based therapies and the challenges that remain in translating these findings into clinical practice. By clarifying the complex interactions between exosomes and macrophages, it hopes to shed light on new therapeutic strategies that could improve outcomes for patients suffering from sepsis-induced ALI.

## 2 Macrophages and sepsis-induced ALI

**Figure 1:**
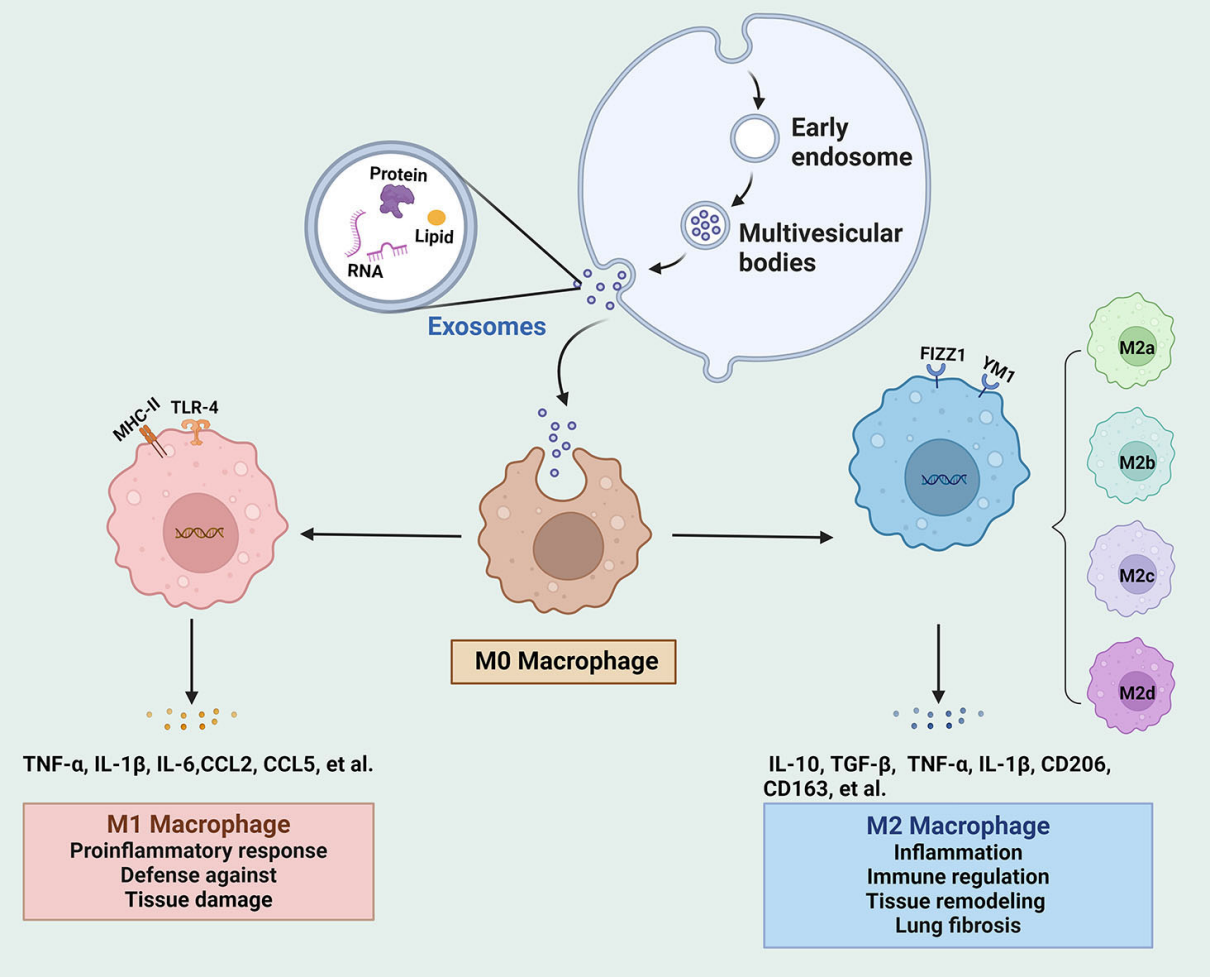
The biogenesis of exosomes and their regulation on the phenotype and function of macrophages. Intraluminal vesicles from multivesicular bodies release exosomes into the extracellular space. Exosomes move to M0 macrophages and modulate the polarization into M1 and M2 under different stimulation conditions. IL, interleukin; mesenchymal stem cells; TLR4, Toll-like receptor 4; TGF, transforming growth factor; TNF, tumor necrosis factor.

Macrophages, acting as key effectors of the innate immune system, are responsible for phagocytosing pathogens, clearing cellular debris, and regulating inflammation (19). In sepsis-related ALI, both alveolar macrophages (AMs) and interstitial macrophages are involved in disease progression. AMs account for the majority of lung-resident macrophages and reside in the alveolar space, which regulate the local immune microenvironment through phagocytosis and the secretion of bioactive molecules (20). Interstitial macrophages, located in the connective tissue surrounding the bronchi, are critical for maintaining lung structure and responding to pathogens (21). During ALI, macrophages exhibit remarkable plasticity, allowing them to polarize into distinct phenotypes in response to microenvironmental cues. The two major polarization states are M1 (classically activated macrophages), and M2 (alternatively activated macrophages) (**Figure 1**) (22). M1 macrophages are pro-inflammatory, which are responsible for initiating the inflammatory response necessary to control infection; however, excessive M1 activation can lead to uncontrolled inflammation and tissue damage (23). Conversely, M2 macrophages are anti-inflammatory that promote the resolution of inflammation and tissue repair (24). The balance between M1 and M2 macrophages is critical in determining the progression of ALI and its subsequent resolution.

In the early phase of sepsis-induced ALI, lung macrophages are activated by pathogens and inflammatory signals, thereby polarizing towards the M1 phenotype to effectively control infection and pathogen clearance (25). Generally, M1 macrophages are activated by several stimulators, such as lipopolysaccharide (LPS) and interferon (IFN)-γ, primarily through the Toll-like receptor 4 (TLR4)/NF-κB signaling pathway (26). These M1 macrophages release large amounts of pro-inflammatory cytokines (e.g., tumor necrosis factor (TNF)-α, interleukin (IL)-1β, and IL-6) and chemokines (e.g., CC-motif chemokine ligand (CCL)2, CCL5), leading to the recruitment of neutrophils and amplifying the inflammatory response (27, 28). Neutrophil-derived enzymes and reactive oxygen species (ROS) further damage alveolar epithelial cells (AECs) and endothelial cells, disrupting the alveolar-capillary barrier, resulting in pulmonary edema and respiratory failure (29). Thus, excessive M1 activation can result in a damaging inflammatory storm, causing alveolar damage and lung dysfunction.

M2 macrophages typically emerge during the later stages of sepsis or during the tissue repair phase (30). According to the induced stimuli and phenotypic features, M2 macrophages are delineated into four subtypes, namely M2a, M2b, M2c and M2d (6). These macrophages show variability in their expressed surface markers, secreted cytokines, and biological roles. M2a macrophages are activated by IL-4, IL-13, and fungal infections, exhibiting elevated levels of CD206, arginase 1, YM1, FIZZ1, and transforming growth factor (TGF)-β, which contribute to pulmonary inflammation and tissue damage. M2b macrophages are stimulated by LPS, IL-1β, and immune complex, and are responsible for the release of pro-inflammatory cytokines such as IL-1β, IL-6, and TNF-α, alongside the anti-inflammatory IL-10, thereby functioning as immunoregulatory macrophages. M2c macrophages are triggered by IL-10, glucocorticoids, and TGF-β, expressing high levels of innate receptors CD206 and CD163, are essential for the tissue remodeling and fibrosis. M2d macrophages are induced by IL-6 and adenosines, which are associated with highly expressed vascular endothelial growth factor and IL-10, participating in processes of angiogenesis and tumor growth (5, 31, 32). During sepsis-induced ALI, M2 macrophages contribute to the resolution of inflammation by promoting extracellular matrix remodeling and cellular regeneration (33). M2 macrophage-derived exosomes inhibit polymorphonuclear neutrophil migration and excessive neutrophil extracellular trap (NET) formation, thereby alleviating lung injury (14).

Given the central role of macrophages in the pathogenesis of sepsis-induced ALI, modulating macrophage polarization has emerged as a promising therapeutic strategy. Exosomes released from different cell types within the lung microenvironment, including alveolar epithelial cells, neutrophils, and MSCs, can influence macrophage polarization. By either inhibiting excessive M1 macrophage activation or enhancing M2 macrophage-mediated anti-inflammatory and reparative functions, exosomes may be possible to reduce inflammatory damage and promote tissue repair in the lungs. Understanding the role of exosomes in regulating macrophage activity during sepsis-induced ALI is essential for developing targeted therapies.

## 3 Biogenesis and function of exosomes

The formation and secretion of exosomes involve several key steps, including endocytosis, multivesicular body formation, and exosome release, which govern the biogenesis of exosomes and the selective incorporation of bioactive molecules into vesicles (11, 34). The biogenesis of exosomes begins with the inward budding of the plasma membrane, creating early endosomes (35). This process, known as endocytosis, is triggered when the cell membrane invaginates to engulf extracellular material, causing the formation of endocytic vesicles that subsequently mature into early endosomes, which are dynamic structures involved in sorting and trafficking of internalized molecules (36). These early endosomes undergo further maturation into late endosomes, also known as multivesicular bodies (MVBs), where invaginations of the endosomal membrane create intraluminal vesicles (ILVs), which contain various biomolecules from the originating cell (37). Eventually, MVBs either fuse with lysosomes for degradation or with the plasma membrane, where they release the ILVs into the extracellular space as exosomes (38). Exosome formation is orchestrated by a variety of molecular machinery, including the endosomal sorting complexes required for transport (ESCRT) pathway, which is responsible for sorting specific proteins into ILVs during exosome biogenesis (39). In addition to the ESCRT-dependent pathway, there are ESCRT-independent mechanisms, such as tetraspanins (e.g., CD63, CD81) and lipids like ceramides, which also contribute to exosome formation (40). These findings indicate that exosome biogenesis is a multifaceted process that can be modulated by the physiological state of the cell and the cellular environment.

Exosomes are enriched with a unique set of proteins, lipids, and nucleic acids, reflecting the physiological or pathological condition of the donor cell (41). Their molecular cargo varies depending on the cell type of origin and the environmental signals received by the cells, making exosomes highly versatile and adaptable intercellular communication tools (42). For instance, exosomes, characterized by the presence of certain proteins such as heat shock proteins and major histocompatibility complex molecules, play roles in antigen presentation and immune modulation (43). Lipids, including sphingomyelin and cholesterol, are abundant in exosomal membranes, contributing to their stability and function in cellular communication (44). Exosomes carrying functional RNAs can regulate gene expression in recipient cells. The selective packaging of these RNAs into exosomes is an active process, controlled by RNA-binding proteins like hnRNPA2B1 and YBX1, which recognize specific RNA sequences or secondary structures, facilitating their inclusion in exosomes (45). This selective sorting mechanism ensures that exosomes deliver targeted regulatory messages to recipient cells, affecting a wide range of biological processes. Thus, exosome-mediated communication by delivering its molecular cargo to recipient cells, plays a vital role in various physiological and pathological processes, including immune response, inflammation, and tissue repair (46).

In the context of sepsis-induced ALI, exosomes have been shown to influence macrophage polarization and function. Exosomes released by immune cells like neutrophils can enhance the inflammatory response by promoting M1 macrophage polarization (16). Conversely, exosomes derived from MSCs and other reparative cells can shift macrophages towards the M2 phenotype, promoting the resolution of inflammation and tissue repair (18). By understanding the molecular mechanisms underlying exosome formation and their interaction with surrounding cells, exosomes could serve as potential therapeutic targets in the management of ALI.

## 4 Exosome-modulated macrophages in sepsis-induced ALI

**Figure 2:**
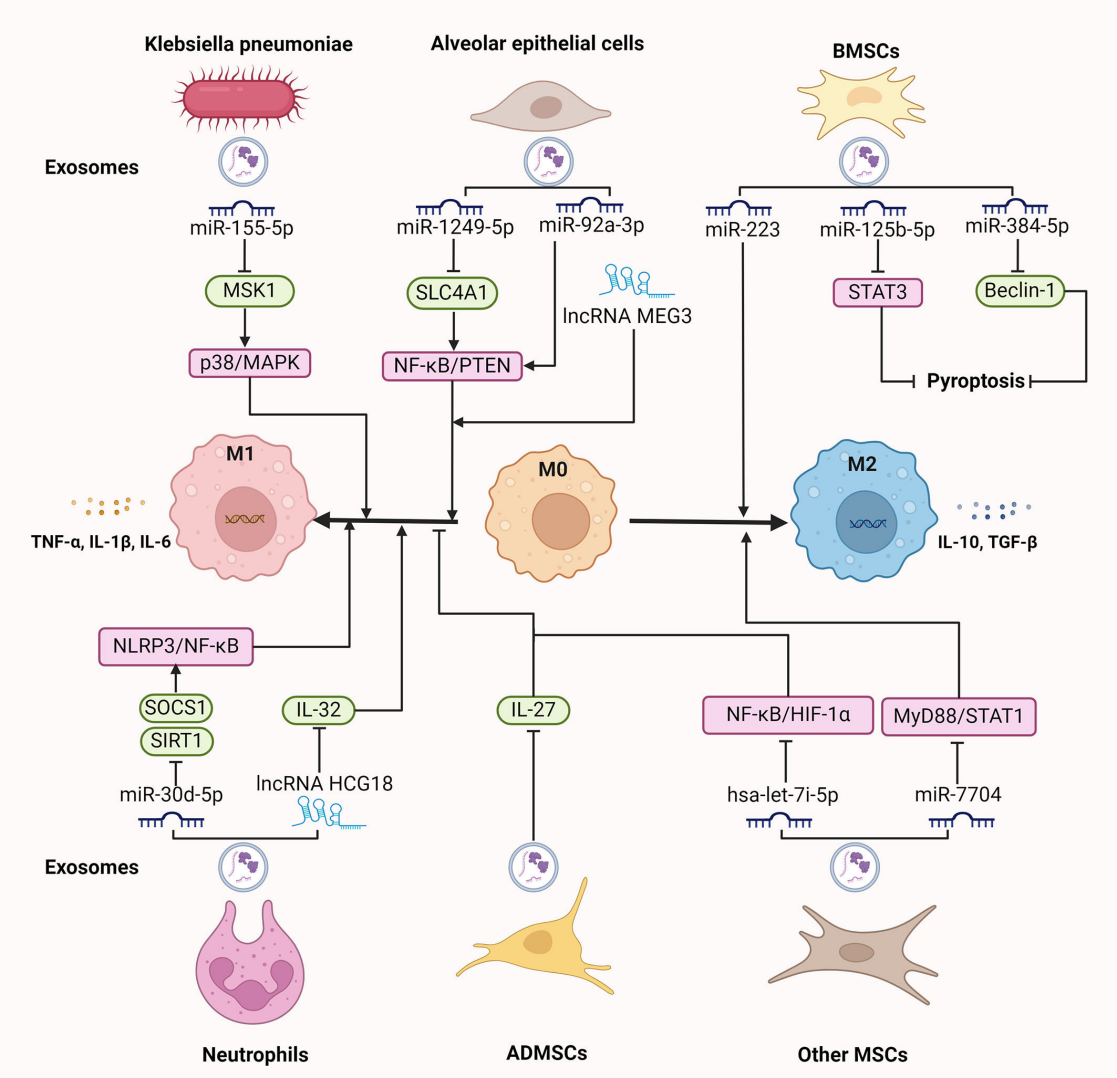
The regulatory role of exosomes on macrophages in sepsis-induced ALI. Exosomal components, like miRNAs and lncRNAs derived from various origins, including Klebsiella pneumoniae, neutrophils, alveolar epithelial cells, and MSCs, regulate macrophage polarization and pyroptosis, participating in the progression of sepsis-induced ALI. ALI, acute lung injury; ADMSCs, adipose-derived mesenchymal stem cells; BMSCs, bone marrow; IL, interleukin; lncRNAs, long non-coding RNAs; miRNAs, microRNAs; MSCs, mesenchymal stem cells; NLRP3, NOD-like receptor 3; STAT3, signal transducer and activator of transcription 3; TLR4, toll-like receptor 4; TGF, transforming growth factor; TNF, tumor necrosis factor. ⊥ indicates an inhibitory effect and → indicates a promoting effect.

Exosomes play a crucial role in modulating macrophage activity during sepsis-induced ALI. These vesicles, released from various cell types in the lung microenvironment, contain proteins, lipids, and nucleic acids that influence macrophage polarization and function, thereby affecting the progression of ALI (**Figure 2**).

### 4.1 Pathogenic germs

Various types of germs, including bacteria and fungi, trigger a cascade of immune responses that can exacerbate lung injury, primarily through the activation of inflammatory pathways and immune cells. The endotoxins from gram-negative bacteria activate inflammatory agents, including complement, neutrophils, and platelets, leading to pulmonary edema and tissue damage (47). Fungus cause ALI characterized by capillary obstruction and interstitial hemorrhage, with the presence of yeast within lung intravascular leukocytes and the transformation to mycelial forms exacerbate lung injury (48). Bacteria-derived exosomes, which carry components such as endotoxins that interact with TLRs on macrophages, elicit neutrophilic pulmonary inflammation along with infiltration of both Th1 and Th17 cells (49). These exosomes trigger the activation of NF-κB signaling pathway, leading to the production of pro-inflammatory cytokines such as TNF-α, IL-6, IL-1β, and IL-8 (50). This robust immune activation by macrophages is essential for the clearance of pathogens, but excessive activation cause uncontrolled inflammation and severe tissue damage in sepsis-induced ALI. It is reported that hypervirulent Klebsiella pneumoniae (hvKp) is highly invasive and pathogenic, and it mediates severe sepsis or septic shock, often accompanied by ALI (51). Recent study has revealed that hvKp-derived exosomes carry high levels of miR-155-5p, which drives macrophage-mediated inflammatory tissue damage and M1 polarization through suppressing the expression of mitogen- and stress-activated kinase 1 and further activating the p38/MAPK signaling pathway (15).

### 4.2 Neutrophils

Neutrophils are rapidly recruited to sites of infection and injury during sepsis (52). In addition to releasing important cytokines, chemokines, and ROS, the formation of NETs that are web-like structures composed of DNA, histones, and proteases, can mediate pyroptosis in alveolar macrophages by regulating NOD-like receptor 3 (NLRP3) deubiquitination, leading to sustained lung inflammation and injury (29). Moreover, neutrophil-derived exosomes are regarded as a new subcellular entity, working as a fundamental link between neutrophil-driven inflammation and lung damage (53). These exosomes carry miRNAs that promote macrophage polarization towards the M1 phenotype, enhancing the production of inflammatory cytokines and chemokines. For example, exosomes from neutrophils under septic conditions contain miR-30d-5p, which inhibits the expression of suppressor of cytokine signaling and sirtuin 1 in macrophages, thereby inducing M1 macrophage polarization and priming macrophage pyroptosis by upregulating NLRP3 inflammasome expression through NF-κB signaling pathway. Whereas, intravenous administration of miR-30d-5p inhibitors reduce the generation of neutrophil-derived exosomal miR-30d-5p, M1 macrophage activation, and macrophage death in the lung (16). Consistently, when alveolar macrophages are co-cultured with TNF-α-stimulated neutrophil-released exosomes, M1 macrophages are activated by exosomal lncRNA HCG18. Further mechanistic evaluation indicated that HCG18 mediates the function of neutrophil-derived exosomes by suppressing the expression of IL-32 in macrophages and thus promotes M1 macrophage polarization. Besides, knockdown of HCG18 in septic mice decreased the M1 macrophage activation, lung macrophage death, and histological lesions (54). These findings suggest that neutrophil-derived exosomes regulate the inflammatory response by targeting pathways involved in macrophage activation and death, which exacerbates lung injury and perpetuates the inflammatory response in sepsis-induced ALI.

### 4.3 Alveolar epithelial cells

AECs are essential for maintaining the structural integrity of the alveoli and regulating the immune response within the lungs. In response to injury or infection, AECs release exosomes that carry signals to neighboring immune cells, including macrophages (55). By constructing co-culture systems of the influenza A virus-induced mouse lung epithelial cells with macrophages, it is found that epithelial cell-derived miR-1249-5p can be delivered into macrophages, which facilitates the release TNF-α and IL-6 in macrophages through repressing the expression of solute carrier family 4 member 1 and thus activating NF-κB signaling pathway (56). Similarly, AEC-derived exosomes contain miR-92a-3p, which increases AM activation and pulmonary inflammation by activating the NF-κB pathway and downregulating PTEN expression; however, inhibition of miR-92a-3p in AECs reduces the pro-inflammatory effects of exosomal miR-92a-3p, highlighting the role of AEC-derived exosomes in exacerbating lung injury (17). In addition, exosomal LncRNA MEG3 from airway epithelial cells is demonstrated to expedite M1 macrophage polarization and pyroptosis (57). These results indicate that exosomal non-coding RNAs derived from AEC act as mediators of intercellular communication, influencing macrophage activity and polarization, contributing to the pathogenesis of sepsis-induced ALI. Moreover, AECs under unresolved endoplasmic reticulum stress release exosomes enriched with tenascin-C, an extracellular matrix glycoprotein, which binds to TLR4 on macrophages. This interaction leads to increased ROS production, mitochondrial damage, which culminates in macrophage pyroptosis via activation of the NF-κB signaling pathway, thus intensifying the inflammatory response in the lungs during sepsis-induced ALI (58).

### 4.4 Mesenchymal stromal cells

MSC-derived exosomes have emerged as promising mediators in alleviating sepsis-induced ALI due to their regenerative, anti-inflammatory, and immunomodulatory properties (39). In the LPS-induced murine ALI model, exosomal miR-7704 from MSCs evokes M2 polarization in lung macrophages by inhibiting the MyD88/STAT1 signaling pathway, thereby restoring pulmonary function and increasing survival (18). Likewise, in a mouse model of cytomegalovirus-induced pneumonia, intravenous administration of mouse MSC-derived exosomes shifts macrophage polarization from the M1 to the M2 phenotype via inactivating the NF-κB/NLRP3 signaling pathway, which reduces the infiltration of inflammatory cells and pulmonary fibrosis (59). Therefore, MSC-derived exosomes help repolarize macrophages toward an anti-inflammatory M2 phenotype, which facilitates tissue repair in sepsis-induced ALI. MSCs from various sources, such as bone marrow, adipose tissue, umbilical cord, and placenta, contribute uniquely to alleviating lung injury through a range of mechanisms.

Bone marrow mesenchymal stem cells (BMSCs) are multipotent stem cells derived from bone marrow and possess immunomodulatory capacity that make them suitable for mitigating inflammatory and immune-mediated conditions like sepsis-induced ALI (60). In LPS-treated alveolar macrophages, BMSC-derived exosomes inhibit M1 polarization and promotes M2 polarization by suppressing cellular glycolysis via downregulating of hypoxia-inducible factor 1α. *In vivo* study further confirmed that these exosomes alleviate the LPS-induced pulmonary inflammation and pathological damage in septic mice (61). Additionally, BMSC-derived exosomal miRNAs exert a protective effect on LPS-induced ALI. Exosomal miR-384-5p from BMSCs relieves LPS-induced autophagy dysfunction in alveolar macrophages by downregulating Beclin-1, which attenuates macrophage viability loss and apoptosis, thus alleviating pulmonary vascular permeability and inflammatory response, and improving the survival rate of ALI rats (62). Similarly, exosomes derived from BMSCs, acting as carriers for delivering miR-125b-5p into macrophages, suppress the expression of signal transducer and activator of transcription 3 (STAT3), thereby halting macrophage pyroptosis and alleviating sepsis-associated ALI (63). Further study unveiled that miR-223 within BMSC-derived exosomes promotes M2 polarization of AMs, which produces anti-inflammatory cytokines like IL-10 and TGF-β, alleviating inflammatory injuries and edema in the lung of LPS-induced ALI rats (64). Thus, BMSC-derived exosomal miRNAs protect against sepsis-induced ALI by modulating macrophage polarization and death. Moreover, BMSC-derived exosomal protein, serum amyloid A1 (SAA1), facilitates LPS internalization by mouse AMs and thus reduces LPS-induced endotoxin, TNF-α, and IL-6 levels, inhibiting lung injury in septic mice (65).

Adipose-derived MSCs (ADMSCs) are pluripotent progenitor cells characterized by their capacity for self-renewal, which ameliorate the immune response and diminish the mortality rates of patients suffering from sepsis by attenuating pro-inflammatory and augmenting anti-inflammatory cytokines, representing one of the most promising stem cells for the treatment of sepsis (66, 67). ADMSC-derived exosomes play a significant role in modulating macrophage phenotypes and functions in sepsis-induced ALI. For example, ADMSC-secreted exosomes can be internalized by LPS-stimulated macrophages and further inhibit the production of IL-27, thereby reducing the release of pro-inflammatory cytokines including IL-6, TNF-α, and IL-1β. ADMSC-derived exosomes also suppress macrophage accumulation in lung tissues and alleviate pulmonary edema and pulmonary vascular leakage, and improve the survival rate of septic mice (68). Of interest, exosomes from ADMSC can transfer mitochondrial components to AMs, improving mitochondrial function and promoting a shift towards the M2 phenotype, as featured with the decreased secretion of IL-1β, TNF-α, and iNOS, as well as enhanced generation of anti-inflammatory cytokines IL-10 and Arg-1. Restoring mitochondrial integrity in LPS-challenged macrophages accelerates oxidative phosphorylation and reduces ROS stress, contributing to the resolution of inflammation (69). Likewise, ADMSC-derived exosomes induce macrophages to secrete TGF-β, which is crucial for promoting M2 polarization and increasing the number of regulatory T cells, thus alleviating sepsis-induced ALI by reducing inflammation and promoting tissue repair (70).

Perinatal MSCs are obtained from various perinatal tissues, like the placenta and umbilical cord, and are valued for their high proliferative capacity and immunomodulatory properties, making them promising candidates for regenerative and therapeutic applications in sepsis-induced ALI (71, 72). Perinatal MSC-derived exosomes modulate macrophage polarization and activity, which is involved in inflammation resolution and tissue repair in ALI. For instance, overexpressing hsa-let-7i-5p in exosomes from human placenta-derived MSCs can reduce M1 polarization and pro-inflammatory cytokine release, along with inactivation of NF-κB and HIF-1α, alleviating tissue edema and leukocyte infiltration in sepsis-induced ALI (73). Consistently, umbilical cord MSC-derived exosomes improve the metabolic function of AMs and facilitate their shift to an anti-inflammatory phenotype, leading to a reduction in LPS-induced ALI (74).

## 5 Exosomes as potential therapeutic targets

Exosomes have garnered interest as potential therapeutic agents due to their ability to modulate immune responses in sepsis-induced ALI. Exosomes serve as crucial mediators of macrophage polarization, playing a dual role in either amplifying or resolving inflammation (**Table 1**). This makes them attractive targets for developing innovative therapies aimed at regulating immune responses, promoting tissue repair, and mitigating the damage caused by excessive inflammation.

**Table 1:** The regulatory role of exosomes on macrophages in sepsis-induced ALI.

Exosomal components	Origin	Targets	Effects	Ref.
Endotoxins	Bacteria	TLRs/NF-κB	Promote pulmonary inflammation, Th1 and Th17 cell infiltration, and pro-inflammatory cytokine release	(80)
MiR-155-5p	Hypervirulent Klebsiella pneumoniae	p38/MAPK	Induce M1 polarization and tissue damage	(15)
MiR-30d-5p	Neutrophils	NF-κB/NLRP3	Promote M1 polarization and macrophage pyroptosis	(16)
LncRNA HCG18	Neutrophils	IL-32	Facilitate M1 polarization and pulmonary damage	(54)
MiR-1249-5p	Alveolar epithelial cells	SLC4A1/NF-κB	Enhance pro-inflammatory cytokine release	(56)
MiR-92a-3p	Alveolar epithelial cells	NF-κB/PTEN	Promote macrophage activation and pulmonary inflammation	(17)
LncRNA MEG3	Alveolar epithelial cells	Undefine	Trigger M1 macrophage polarization and pyroptosis	(81)
Tenascin-C	Alveolar epithelial cells	TLR4/NF-κB	Promote ROS production, mitochondrial damage, and macrophage pyroptosis	(58)
miR-7704	MSCs	MyD88/STAT1	Induce M2 polarization and restore pulmonary function	(18)
Undefine	MSCs	NF-κB/NLRP3	Mitigate inflammatory cell infiltration and pulmonary fibrosis	(59)
Undefine	BMSCs	HIF-1α	Alleviate pulmonary inflammation and tissue damage	(61)
MiR-384-5p	BMSCs	Beclin-1	Inhibit autophagy dysfunction and pulmonary inflammation	(62)
MiR-125b-5p	BMSCs	STAT3	Reduce macrophage pyroptosis	(63)
MiR-223	BMSCs	Undefine	Promotes M2 polarization and alleviate inflammatory injuries and edema in the lung	(64)
Serum amyloid A1	BMSCs	Undefine	Inhibit pro-inflammatory cytokine release and lung injury	(65)
Undefine	ADMSCs	IL-27	Alleviate pulmonary edema and pulmonary vascular leakage	(82)
Mitochondrial components	ADMSCs	Undefine	Restore mitochondrial integrity in macrophages	(69)
Undefine	ADMSCs	Undefine	Promote M2 polarization and tissue repair	(70)
Hsa-let-7i-5p	Perinatal MSCs	NF-κB, HIF-1α	Reduce M1 polarization and pro-inflammatory cytokine release	(73)
Undefine	Umbilical cord MSC	Undefine	Improve the metabolic function of macrophages and facilitate their anti-inflammatory functions	(74)

One of the most promising therapeutic applications of exosomes lies in their ability to carry anti-inflammatory molecules that can modulate the immune reaction. As concluded above, MSC-derived exosomes have emerged as potent candidates for treating sepsis-induced ALI. These exosomes carry a range of bioactive molecules, including miRNAs, lncRNAs, and proteins, which have demonstrated the capacity to suppress excessive immune activation and promote tissue repair (75). Preclinical studies using MSC-derived exosomes in animal models of sepsis-induced ALI have shown promising results. For instance, the administration of MSC-derived exosomes has been found to reduce M1 macrophage polarization and inflammatory cytokine production; meanwhile, they facilitate the polarization of macrophages towards the M2 phenotype, which contributes to resolving inflammation and promoting tissue repair (76). Besides, by decreasing the levels of exosomal miRNA-146a derived from lung epithelial cells, the natural agent salidroside can reduce the expression of pro-inflammatory factors by inactivating the TLR4-mediated NF-kB signaling pathway, exerting a protective effect in sepsis-induced ALI (77). Of importance, exosomes are smaller, less immunogenic, and easier to administer than whole cells, and they can be engineered to carry specific therapeutic cargoes that target key pathways involved in inflammation and tissue repair. Their ability to cross biological barriers, such as the alveolar-capillary barrier, makes them well-suited for delivering therapeutic molecules to the lungs (11). It is reported that a novel exosome-based drug is produced by engineering modification of umbilical cord MSC-derived exosomes that loaded with anti-PD-1 peptide, and it reduces the expression levels of pro-inflammatory cytokine and the apoptosis of lung cells, as well as increases the expression of anti-inflammatory cytokine IL-10 and the ratio of M2/M1 macrophage, thereby attenuating the inflammatory response in septic mice (78). Specific surface markers on macrophages can be targeted by engineered exosomes that deliver drugs and modulate immune responses to enhance their therapeutic efficacy. For example, CD206 is a marker for M2 macrophages, which are associated with tumor progression and immune suppression. Engineered exosomes that loaded with chemotherapeutic agents can strengthen antitumor immunity and reduce tumor burden by targeting CD206-positive M2 macrophages (79). In this content, engineered exosomes offer the potential to enhance the specificity and efficacy of treatments by delivering targeted interventions to macrophages involved in sepsis-induced lung injury. This approach could also be used in combination with other treatments, such as antibiotics and immunomodulatory agents, to enhance the overall therapeutic outcome.

While exosome-based therapies hold promise for treating sepsis-induced ALI, several challenges remain. These include optimizing the isolation and production of exosomes on a large scale, ensuring the stability and efficacy of exosomal cargo, and addressing potential off-target effects. Additionally, clinical trials are needed to validate the safety and efficacy of exosome-based therapies in patients with sepsis-induced ALI, as well as investigate the optimal timing of intervention, whether before or after the onset of sepsis. Future research should focus on further understanding the mechanisms of exosome-mediated immune modulation, refining engineering techniques for exosomes, and exploring the potential of combination therapies that leverage exosomes alongside traditional treatments.

## 6 Conclusion and perspective

Macrophages are involved in the progression of ALI, acting as both drivers of inflammation and facilitators of tissue repair. The recent recognition of exosomes as key modulators of macrophage function opens new avenues for understanding the molecular mechanisms underlying the immune response in sepsis-induced ALI. Exosomes play a crucial role in determining the balance between pro-inflammatory M1 macrophages and anti-inflammatory M2 macrophages during sepsis-induced ALI. Their ability to carry and deliver specific miRNAs, proteins, and lipids to macrophages makes them essential mediators of immune responses. Exosomes derived from pathogens, neutrophils, and alveolar epithelial cells, tend to promote M1 macrophage polarization, perpetuating inflammation and exacerbating lung injury. In contrast, MSC-derived exosomes have been shown to promote M2 macrophage polarization, facilitating the resolution of inflammation and promoting tissue repair. This dynamic modulation of macrophage polarization by exosomes suggests that the fine-tuning of exosome production and cargo composition could be an effective therapeutic strategy. By inhibiting the pro-inflammatory exosomes or by upregulating the anti-inflammatory exosomes, it may be possible to shift the balance in favor of M2 macrophages, promoting healing and reducing inflammation in sepsis-induced ALI. Preclinical studies have demonstrated the ability of MSC-derived exosomes to reduce inflammation, promote tissue repair, and improve survival in animal models of ALI. However, translating these promising findings into clinical practice presents several challenges. The production, isolation, and purification of exosomes at a scale sufficient for therapeutic use remain significant hurdles. Moreover, ensuring the stability and targeted delivery of exosomes to the lungs requires further technological advancements. The development of engineered exosomes, designed to carry specific therapeutic cargoes such as miRNAs and proteins, offers an exciting possibility for enhancing the specificity and efficacy of exosome-based therapies. These engineered exosomes could be tailored to target specific macrophage populations or lung cells, ensuring that their therapeutic effects are maximized while minimizing off-target effects. Future research should focus on refining exosome-based interventions and exploring combination therapies in clinical settings. Furthermore, it should be noted that exosomes derived from different cell types or released under different conditions can carry vastly different cargoes, with distinct effects on target cells. Understanding how the microenvironment influences exosome biogenesis and cargo selection is essential for designing effective exosome-based therapies.
